# Dorsal Approach in the Surgical Treatment of Complex Dorsal Dislocation of Index Metacarpophalangeal Joint; a Case Report 

**DOI:** 10.22037/aaem.v10i1.1479

**Published:** 2022-02-09

**Authors:** Shahab Aldin Sattari, Ali Reza Sattari, Kamran Heydari, Seyed Matin Sadat Kiaei, Farshad Zandrahimi, Mehdi Mohammadpour

**Affiliations:** 1 Department of Neurosurgery, Johns Hopkins University School of Medicine, Baltimore, Maryland, United States.; 2 Department of Surgery, MedStar Health Baltimore, Baltimore, Maryland, United States.; 3 Skull base research center, Loghman Hakim Hospital, Shahid Beheshti University of Medical Science, Tehran, Iran.; 4 Bone and Joint Reconstruction Research Center, Department of Orthopedics, School of Medicine, Iran University of Medical Sciences, Tehran, Iran.; 5 Department of Orthopedic, School of Medicine, Shahid Bahonar Hospital, Kerman University of Medical Sciences, Kerman, Iran.

**Keywords:** Hand injuries, metacarpophalangeal joint, metacarpal bones, wounds and injuries, fractures, bone, joint dislocations

## Abstract

Complex metacarpophalangeal (MCP) joint dislocation is an uncommon entity, which occurs following a hyperextension injury. Closed reduction is not feasible due to entrapped volar plate and/or coexisting fractures. Various approaches and techniques have been proposed for treatment of complex MCP dislocation; however, controversies exist over which one is superior. This study describes a right-handed 14-year-old boy who fell on the outstretched hand and sustained a dorsal dislocation of the left index MCP joint. The dislocation was complicated by an epiphyseal metacarpal head fracture with dorsal-ulnar displacement of the osteochondral fragment. The patient underwent open reduction through the dorsal approach, and the metacarpal head was fixed via the two-screw technique. The patient resumed left-hand function after six weeks. At the two-year follow-up, the range of motion and grip strength were normal, the patient was pain-free, and no sign of growth disturbance or joint stiffness was detected. Dorsal surgical approach with screw fixation is a feasible technique for the treatment of complex MCP dislocation, especially when it is complicated by a large epiphyseal head fracture.

## 1. Introduction:

Hand trauma is one of the most common orthopedic emergencies (1), of which interphalangeal joint sprain, phalangeal, and metacarpal fractures are common (2). However, the metacarpophalangeal (MCP) joint dislocation is rare (3). Typically, MCP joint dislocation occurs due to hyperextension injury following a fall on the outstretched hand. The dislocation can be classified as a volar or dorsal (4), and further as a simple, which is reducible, or complex, which requires open reduction due to entrapped volar plate, and/or concomitant fracture (2,4). Generally, two surgical approaches have been proposed with proponents on either side. The volar approach gives direct access to the lesion while carrying the risk of neurovascular injury. Conversely, the dorsal approach enables the surgeon to repair the osteochondral defect with a lower probability of neurovascular damage (5). The best approach for each patient is still a matter of debate, and it largely depends on the dislocation characteristics, surgeon’s experience, and preference (6). Hereby, we describe the dorsal approach for the treatment of a rare case of complex dorsal dislocation of the index MCP joint with entrapped volar plate and large epiphyseal metacarpal head fracture. 

## 2. Case presentation:

**Figure 1:**
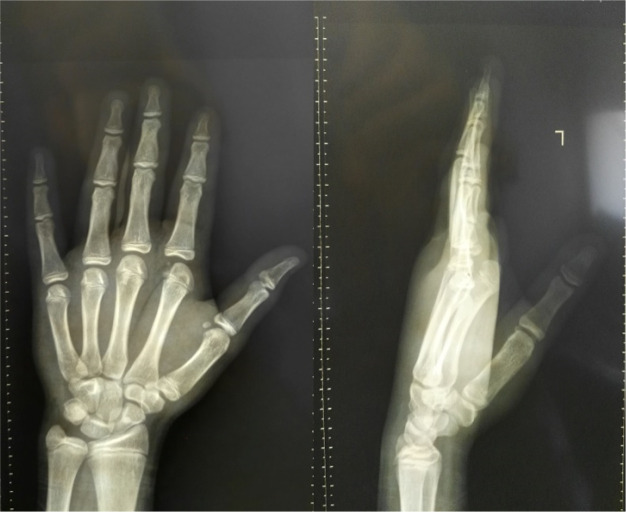
Left hand anteroposterior (left) and lateral (right) radiographs preoperatively demonstrate index metacarpophalangeal joint space widening, index metacarpal epiphyseal head fracture, and dorsal-ulnar displacement of the osteochondral fragment

 A 14-year-old boy presented to the orthopedic emergency department due to left-hand trauma following a fall on the outstretched hand. On physical examination, the left index MCP joint was swollen, deformed, and tender to palpation. The MCP joint’s active range of motion was restricted to 0-20º (versus the contralateral MCP joint’s active range of motion of 10º extension to 80º flexion). The left-hand x-ray revealed 2nd MCP joint dorsal dislocation and epiphyseal metacarpal head fracture with dorsal-ulnar displacement of the osteochondral segment (figure 1). 

**Figure 2:**
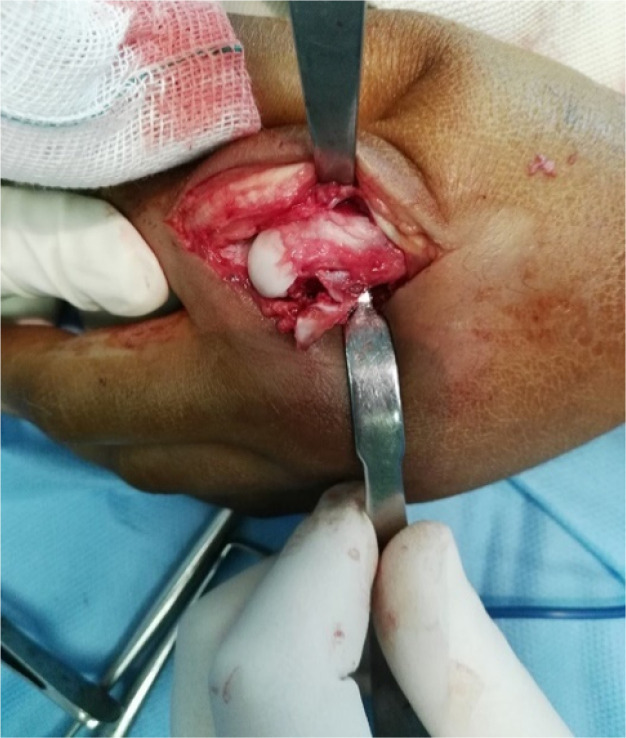
Dorsal Approach, index metacarpal head fracture associated with the osteochondral fragment

**Figure 3:**
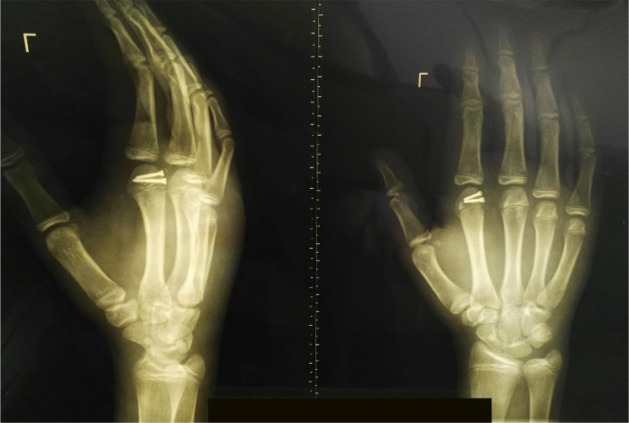
Two-year post-operation left hand oblique (right) and anteroposterior (left) radiographs

He underwent open reduction through a dorsal approach. A small curvilinear incision was made over the dorsal aspect of the joint using scalpel #11. The extensor mechanism was split, and the branch of the neurovascular bundle was identified and retracted using Farabuef (figure 2), and then the joint capsule was split longitudinally. The entrapped volar plate was identified and reinserted using a 4-0 Vicryl suture, and then the osteochondral fragment was reduced and fixed using two #2 screws. Fluoroscopy confirmed joint reduction, and then the joint capsule, extensor mechanism, subcutaneous tissue, and skin were closed with appropriate suture, and the hand was immobilized using a short volar splint. After two days, the intermittent joint motion was started, and after two weeks, control radiography was taken, and the splint was removed. At six weeks post-operation, the patient resumed a range of motion of 10º extension to 70º flexion with mild stiffness. At the two-year follow-up, the range of motion and grip strength were normal, the patient was pain-free, and no sign of growth disturbance or joint stiffness was detected (figure 3). 

## 3. Discussion:

MCP joint dislocation is uncommon relative to other hand injuries. While simple dislocation is reducible with closed technique, the complex dislocation requires open reduction because of interposing soft tissue and/or concomitant fracture (2,7,8). The most common interposing soft tissue is the volar plate, in which the hyperextension ruptures the volar plate at its weakest point (i.e., membranous insertion to metacarpal periosteum), and while the metacarpal head displaces down palmary, the volar plate lodges into the joint space (7). 

Timely surgical intervention is crucial in the management of complex MCP joint dislocation, as the longer the joint remains displaced, the higher the risk of complications such as osteonecrosis, growth arrest, restricted range of motion, and arthritis (9). To date, several approaches and techniques have been proposed, and controversies exist over which one is superior. The volar approach provides the surgeon with direct access to the lesion, tendon, ligament, and volar plate. However, the risk of neurovascular injury is considerable. By using the volar approach, radial neurovascular bundles are more prone to injury in the index and middle finger, whereas ulnar neurovascular bundles are more at risk in the ring and little fingers. Of note, the dorsal approach not only provides adequate exposure but also allows the surgeon to repair the osteochondral segment and bypasses the neurovascular injury risk associated with the volar approach (4,5).

Either screw or Kirschner wire (K-wire) can be used as a fixator. However, K-wire requires a longer immobilization time and is not appropriate in case of large, fragmented, or distal fractures. K-wire may impinge on periarticular and articular surfaces and restrict the range of motion (7).

Pereira et al. reported a lateral approach in the treatment of a 16-year-old male harboring complex MCP dislocation with a small osteochondral fragment (10). Although the lateral approach allows access to volar and dorsal structures and lowers the risk of surgical scarring, the risk of neurovascular injury is higher than the dorsal approach, and its efficacy is yet to be evaluated in the case of large osteochondral fragments. Kodama et al. used arthroscopic reduction for an 11-year-old boy with complex dislocation of index MCP joint due to entrapped volar plate (11). This minimally invasive technique lowers the risk of neurovascular injury; however, it is limited in case of concomitant fracture. Boden et al. described an 8-year-old girl with complex dislocation of the index MCP joint. The dislocation was reduced using the hook technique, in which the hook was inserted at the site of skin dimpling over the proximal palmar crease (characterizing as “puckering sign”), and then traction was applied to the volar structures (12). Sodha et al. used the percutaneous dorsal stab technique for the treatment of four cases of complex MCP dislocation, in which a dorsal incision was directed through the dorsal retinaculum and joint capsule into the dorsum of the metacarpal neck. Then, the volar plate was divided, and reduction was achieved (13). Although the percutaneous technique is safe, time-saving, and feasible, it cannot be used in case of concurrent fracture.

## 4. Conclusion:

The dorsal approach with screw fixation is a feasible and successful technique in the case of dorsal complex MCP dislocation, especially when it is complicated by large metacarpal head fracture.

## 5. Declarations:

### 5.1 Acknowledgment

None.

### 5.2 Authors' contribution

All authors contributed to the study conception and design. All authors read and approved the final manuscript.

Conceptualization: Mehdi Mohammadpour, Shahab Aldin Sattari, Kamran Heydari, Farshad Zandrahimi

Writing draft, editing and preparation: Shahab Aldin Sattari, Mehdi Mohammadpour, Ali Reza Sattari, Seyed Matin Sadat Kiaei

### 5.3 Funding and Support

The authors did not receive support from any organization for the submitted work.

### 5.3 Conflict of Interest

The authors have no conflicts of interest to declare that are relevant to the content of this article.

### 5.4 Data Availability

Author(s) guarantee that data of the study are available and will be provided if anyone needs them.

### 5.5 Ethics approval

 All procedures performed in studies involving human participants were in accordance with the ethical standards of the institutional and/or national research committee and with the 1964 Helsinki Declaration and its later amendments or comparable ethical standards. Informed consent was obtained from the guardians of the participant included in the study.

## References

[1] Kreutz-Rodrigues L, Gibreel W, Moran SL, Carlsen BT, Bakri K (2022). Frequency, Pattern, and Treatment of Hand Fractures in Children and Adolescents: A 27-Year Review of 4356 Pediatric Hand Fractures. Hand (N Y)..

[2] Dinh P, Franklin A, Hutchinson B, Schnall SB, Fassola I (2009). Metacarpophalangeal Joint Dislocation. J Am Acad Orthop Surg [Internet]..

[3] Calfee RP, Sommerkamp TG (2009). Fracture-dislocation about the finger joints. J Hand Surg Am..

[4] Barry K, McGee H, Curtin J (1988). Complex dislocation of the metacarpo-phalangeal joint of the index finger: a comparison of the surgical approaches. J Hand Surg Br..

[5] Sumarriva G, Cook B, Godoy G, Waldron S (2018). Pediatric complex metacarpophalangeal joint dislocation of the index finger. Ochsner J..

[6] Lee J-K, Jo Y-G, Kim J-W, Choi YS, Han S-H (2017). Open reduction and internal fixation for intraarticular fracture of metacarpal head. Orthopade..

[7] Light TR, Ogden JA (1988). Complex dislocation of the index metacarpophalangeal joint in children. J Pediatr Orthop..

[8] Türker T, Sheppard JE (2015). Emergency Open Reduction for an Irreducible Dislocation of the Metacarpophalangeal Joint of the Thumb in a Child. J Hand Microsurg..

[9] Rubin G, Orbach H, Rinott M, Rozen N (2016). Complex dorsal metacarpophalangeal dislocation: long-term follow-up. J Hand Surg Am..

[10] Pereira JM, Quesado M, Silva M, Carvalho JDD, Nogueira H, Alves J (2019). The Lateral Approach in the Surgical Treatment of a Complex Dorsal Metacarpophalangeal Joint Dislocation of the Index Finger. Case Rep Orthop..

[11] Kodama A, Itotani Y, Mizuseki T (2014). Arthroscopic reduction of complex dorsal metacarpophalangeal dislocation of index finger. Arthrosc Tech..

[12] Boden RA, Cavale N, Fleming ANM (2006). Kaplan’s puckering sign in complex dorsal dislocation of the metacarpophalangeal joint of the index finger. J Hand Surg Br..

[13] Sodha S, Breslow GD, Chang B (2004). Percutaneous technique for reduction of complex metacarpophalangeal dislocations. Ann Plast Surg..

